# Phase II Study of Concurrent Capecitabine and External Beam Radiotherapy for Pain Control of Bone Metastases of Breast Cancer Origin

**DOI:** 10.1371/journal.pone.0068327

**Published:** 2013-07-10

**Authors:** Yulia Kundel, Nicola J. Nasser, Ofer Purim, Rinat Yerushalmi, Eyal Fenig, Raphael M. Pfeffer, Salomon M. Stemmer, Shulamith Rizel, Zvi Symon, Bella Kaufman, Aaron Sulkes, Baruch Brenner

**Affiliations:** 1 Institue of Oncology, Davidoff Cancer Center, Rabin Medical Center, Beilinson Campus, Petach Tikva, Israel; 2 Institute of Oncology, Chaim Sheba Medical Center, Tel Hashomer, Israel; 3 Sackler Faculty of Medicine, Tel Aviv University, Tel Aviv, Israel; University Clinic of Navarra, Spain

## Abstract

**Background:**

Pain from bone metastases of breast cancer origin is treated with localized radiation. Modulating doses and schedules has shown little efficacy in improving results. Given the synergistic therapeutic effect reported for combined systemic chemotherapy with local radiation in anal, rectal, and head and neck malignancies, we sought to evaluate the tolerability and efficacy of combined capecitabine and radiation for palliation of pain due to bone metastases from breast cancer.

**Methodology/Principal Findings:**

Twenty-nine women with painful bone metastases from breast cancer were treated with external beam radiation in 10 fractions of 3 Gy, 5 fractions a week for 2 consecutive weeks. Oral capecitabine 700 mg/m^2^ twice daily was administered throughout radiation therapy. Rates of complete response, defined as a score of 0 on a 10-point pain scale and no increase in analgesic consumption, were 14% at 1 week, 38% at 2 weeks, 52% at 4 weeks, 52% at 8 weeks, and 48% at 12 weeks. Corresponding rates of partial response, defined as a reduction of at least 2 points in pain score without an increase in analgesics consumption, were 31%, 38%, 28%, 34% and 38%. The overall response rate (complete and partial) at 12 weeks was 86%. Side effects were of mild intensity (grade I or II) and included nausea (38% of patients), weakness (24%), diarrhea (24%), mucositis (10%), and hand and foot syndrome (7%).

**Conclusions/Significance:**

External beam radiation with concurrent capecitabine is safe and tolerable for the treatment of pain from bone metastases of breast cancer origin. The overall and complete response rates in our study are unusually high compared to those reported for radiation alone. Further evaluation of this approach, in a randomized study, is warranted.

**Trial Registration:**

ClinicalTrials.gov NCT01784393NCT01784393

## Introduction

Painful metastatic bone disease is a common complication of breast cancer. Systemic treatments, such as chemotherapy, hormonal therapy, and targeted biological therapies, may be effective, but the time to response ranges from several weeks to months. Therefore, to achieve rapid control of pain, local radiotherapy is frequently utilized as a bridge, initiated either before the onset of chemotherapy or in combination with hormonal therapy [1].

To optimize pain control, researchers have attempted to modulate radiation dose intensities and treatment schedules. In a large national study conducted between 1974 and 1980, the Radiation Therapy Oncology Group (RTOG) tested the effectiveness of five dose fractionation schedules for palliation of symptomatic bone metastases [2]. Patients with isolated lesions were randomly assigned to treatment with 40.5 Gy in 15 fractions or 20 Gy in 5 fractions; patients with multiple metastases were assigned to treatment with 30 Gy in 10 fractions, 15 Gy in 5 fractions, 20 Gy in 5 fractions, or 25 Gy in 5 fractions. Although almost all patients experienced some relief within 4 weeks, in about half of them the time to complete relief was longer. There were no significant differences in duration of pain relief among the different arms, and all treatment dose schedules were equally effective [2]. On reanalysis of the RTOG data, Blitzer [3] concluded that the more protracted schedules resulted in improved pain relief. In a subsequent trial, Price et al. [4] randomized 288 patients to receive either 8 Gy in one fraction or 30 Gy in 10 daily fractions; no differences were found in the probability of attaining pain relief, speed of onset of relief, or duration of relief.

The first report of a dose-response relationship in pain control in this setting was reported by the RTOG using pooled data from published phase III trials [5]. To compare the different study arms, the biological effective dose (BED) was calculated for each schedule. Regression analysis yielded a statistically significant increase from 1.00 to 3.32 in the odds ratio with an increase in BED from 14.4 Gy to 51.4 Gy.

Over the last several decades, concurrent chemotherapy and radiation has been used successfully in a variety of malignancies [6]–[10], and it has become the mainstay of treatment for anal [10] and rectal cancers [11]. The Gastrointestinal Tumor Study Group (GITSG) reported a significant survival advantage for patients receiving radiation with bolus 5-fluorouracil (5FU) following curative resection of pancreatic cancer [12], [13]. Improved survival with concurrent 5FU-containing chemoradiation regimens has also been shown in esophageal [14], head and neck [15], and cervical cancers [16].

Capecitabine is an oral prodrug of 5FU which acts as a radiosensitizer, enhancing the BED of radiation. It is being increasingly used with radiation instead of 5FU owing to its ease of administration [17]–[25]. In a single institution study of 32 patients with gastrointestinal malignancies, Vaishampayan et al. [17] reported very little toxicity with concurrent radiotherapy and capecitabine at a median dose of 1600 mg/m^2^/day (5 days a week). These findings were supported in phase II and III studies of rectal cancer wherein chemoradiation with capecitabine 1650 mg/m^2^/d for 14 days was found to be safe and well tolerated [18], [26].

However, all of these studies focused on localized malignancies, and information on combined chemotherapy and radiation for palliation of pain from bone metastases, including those of breast cancer origin, is lacking. Hence, the aim of the present phase II prospective clinical trial was to test the effectiveness and safety of treatment with localized external beam radiotherapy and concurrent capecitabine in patients with painful bone metastases from breast cancer.

## Methods

The protocol for this trial and supporting CONSORT checklist are available as supporting information; see Checklist S1 and Protocol S1.

### Ethics Statement

The study was approved by the Ethics Committees of Rabin Medical Center and Chaim Sheba Medical Center. Written informed consent was obtained from each participant prior to study entry. ClinicalTrials.gov Identifier: NCT01784393.

### Eligibility

The study group consisted of women attending two tertiary cancer centers in Israel from May 2004 to April 2007. Eligibility criteria for the study were age 18 years or older, histologically confirmed breast cancer, radiographic evidence of bone metastases on radionuclide bone scan, computerized tomography (CT) or magnetic resonance imaging, and pain corresponding to the bone lesions. Patients with 1–3 metastatic bone lesions were enrolled, regardless of prior therapy. Bone biopsy was performed according to the discretion of the treating physician.

### Radiation Therapy

Radiation fields were designed to cover all metastatic bone regions using CT simulation. A dose of 30 Gy was delivered in 10 fractions of 3 Gy at photon beam energy of 6/18 MV, 5 days per week, mainly in anterior-posterior or posterior-anterior fields, 1–3 fields per bone metastasic site.

### Chemotherapy

Oral capecitabine 1400 mg/m^2^ was administered 5 days per week, in 2 divided daily doses, concurrently with radiotherapy. Capecitabine was administered as 500 mg tablets only, to limit the risk of confusion, and the total daily dose was therefore rounded to the nearest 500 mg This dose is slightly lower than those used in an earlier study of concurrent treatment of rectal carcinoma which is usually 1650 mg/m^2^ per day [18]. We reduced the dose by 15% to 1400 mg/m^2^ for safety reasons, due to lack of previous studies in breast cancer combining capecitabine and radiation.

### Clinical Evaluation

Patients were evaluated for toxicity once weekly during treatment and every 4 weeks thereafter, until 12 weeks after the completion of chemoradiation. Toxicity was graded according to the NCI common terminology criteria for adverse events (CTCAE) version 3 [27]. Patients were asked to score their pain on a scale of 0 (no pain) to 10 (worst possible pain) before treatment and at 1, 2, 4, 8 and 12 weeks after treatment initiation. Pain intensity was assessed utilizing a 5 steps ladder ranging from 0 to 4; 0 points denotes no pain, 4 points denotes strong pain. A score of 1–4 was defined as mild pain, 5–6 moderate pain, 7–8 severe pain, 9–10 strong pain [28]. Side effects were graded for intensity on a scale of 0 (no side effects) to 4 (severe side effects interfering with activities of daily living). Consumption of analgesics was evaluated by the physician on the basis of the medical records using the 5-point WHO score, as follows: level 0, no analgesics required; level 1, non-narcotic analgesics required occasionally; level 2, non-narcotic analgesics required regularly; level 3, narcotic analgesics required occasionally; level 4, narcotic analgesics required regularly.

### Response to Treatment

Response was assessed, as recommended by the International Bone Metastases Consensus [29], at 1, 2, 4, 8 and 12 weeks after treatment initiation. The following definitions were applied: complete response (CR) - pain score of 0 and no increase in analgesics consumption; partial response (PR) - decrease of at least 2 points in pain score without an increase in analgesics consumption; stable pain (SP) - change of 1 point or no change in pain score with no change in analgesics consumption; progressive pain (PP) - increase of 2 or more points in pain score.

### Statistical Analysis

Sample size was calculated using Simon’s optimal two-stage method for *P*_0_ = 0.05 and *P*_1_ = 0.25, using error probability limits *α* = 0.05 and *β* = 0.20. It was estimated that at least 17 assessable patients were required. An interim analysis was planned to stop the trial in case less than one response, or any grade 4 or 5 toxicities, were observed among the first 9 treated patients.

Results are reported as mean ± standard deviation (SD). Statistical analysis was performed using the student T-test, and the statistical soft ware programs Microsoft Office Excel® 2007 and GraphPad Prism version 4.1.

## Results

### Clinical Characteristics

**Figure 1:**
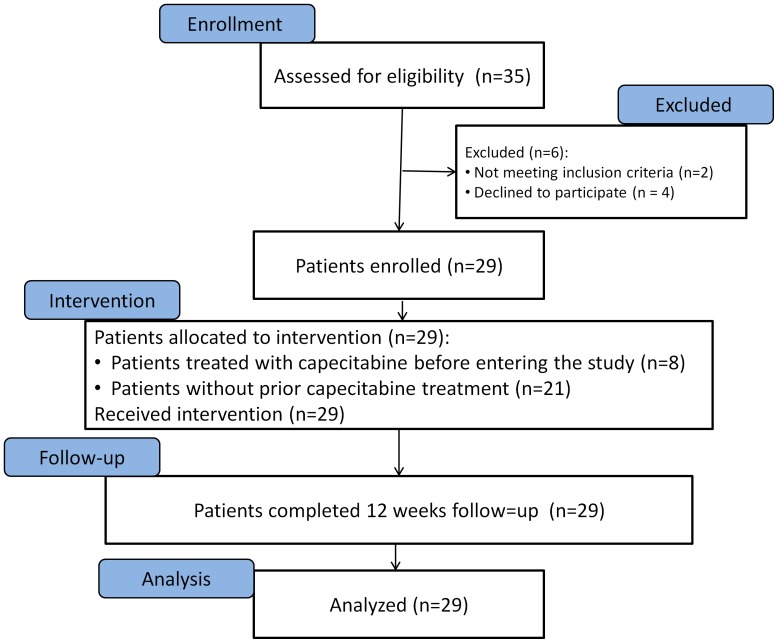
Flow chart of study selection process.

Twenty-nine patients were included in the study. Their main clinical characteristics are summarized in Table 1. Median age was 59 years (range, 35–84 years). The mean number of bone metastatic lesions was 1.3 (range, 1–3). Besides the bone metastases, metastatic lesions were found in the brain in one patient and in the liver in 6 patients. Eight patients (28%) were receiving capecitabine for treatment of the metastatic disease at the time of enrollment to the study; in these cases, the capecitabine dose was adjusted to 1400 mg/m^2^/day, in 2 divided doses, during the radiation days (see METHODS). Eleven patients (38%) were also treated with bisphosphonates during chemoradiation. All patients had estrogen receptor positive tumors. In one patient the tumor was HER2 positive. All 29 patients completed the study protocol (Figure 1). After completing the study protocol, patients were treated according to the discretion of the treating physician.

**Table 1:** Characteristics of 29 patients with painful bone metastases of breast cancer origin and the treatment fields.

Patient	Age (Yr.)	Bone metastases treatedwith radiation	Extraosseous metastasis	Bisphosphonates use	Radiation Fields
1	54	Hip		No	AP-PA
2	76	C1–3,Rt. Ischium, Pubis		No	LAT, AP-PA
3	52	Rt. Hemipelvis		No	AP-PA
4	56	Hip		No	AP-PA
5	51	Shoulder, L1–L5		No	AP-PA,PA
6	84	D4 - L1	Liver	No	PA
7	77	L3 - S2	Liver	No	PA
8	54	Lt. Femur	Brain	No	AP-PA
9	46	Rt Femur, Lt Femur		Yes	AP-PA
10	35	D9 - L1, L3 - L5		No	PA
11	51	Rt. Hemipelvis		Yes	AP-PA
12	62	Lt. Hemipelvis	Liver	No	AP-PA
13	69	Lt. Femur		No	AP-PA
14	55	Rt.Hemipelvis		No	AP-PA
15	60	Sacrum		Yes	AP-PA
16	57	C6–C8		Yes	PA
17	73	Sacrum, Rt. Femur, D6–D9		No	PA,AP-PA,PA
18	78	Rt. Femur, Acetabulum	Chest wall mass	No	AP-PA
19	78	Pelvis	Liver	Yes	AP-PA
20	41	Rt. Acetabulum		No	AP-PA
21	37	Rt. Humerus	Liver	No	AP-PA
22	49	D9–D12		Yes	PA
23	63	Rt. Hemipelvis		Yes	AP-PA
24	59	L3 - Sacroiliac joint		Yes	PA
25	60	Sacrum, Rt. Hemipelvis		Yes	AP-PA
26	62	C6 - D3	Liver	Yes	PA
27	54	D3 - D9		Yes	PA
28	74	L1– Sacroiliac joint		No	PA
29	64	D5- C4		No	PA

Abbreviations: PA – Postero Anterior; AP- Antero Posterior, Lat- lateral fields.

### Toxicity

Treatment-related toxicity, according to the NCI CTCAE version 3, is depicted in Table 2. Chemoradiation was generally well tolerated and side effects were mild (grade 1 or 2). Grade 2 side effects included nausea in 2 patients (7%), weakness in one patient (3%) and radiation dermatitis in one patient (3%). Grade 1 side effects included mainly nausea in 9 patients (33%), diarrhea in 7 (24%), weakness in 6 (21%), mucositis in 3 (10%), and hand and foot syndrome in 2 (7%). All toxicities completely resolved within 2 weeks from completion of treatment.

**Table 2:** Treatment related side effects.^1^

Side effects^2^	Grade I	Grade II	Grade III/IV
Diarrhea	7 (24%)	0	0
Hand foot syndrome	2 (7%)	0	0
Mucositis	3 (10%)	0	0
Nausea	9 (31%)	2 (7%)	0
Weakness	6 (21%)	1 (3%)	0
Radiation dermatitis	1 (3%)	1 (3%)	0

1 Number of patients experiencing the toxicity and their percentage.

2 Side effects were graded for severity according to the NCI common terminology criteria for adverse events (CTCAE) vertion 3.

### Pain and Analgesics Scores

**Figure 2:**
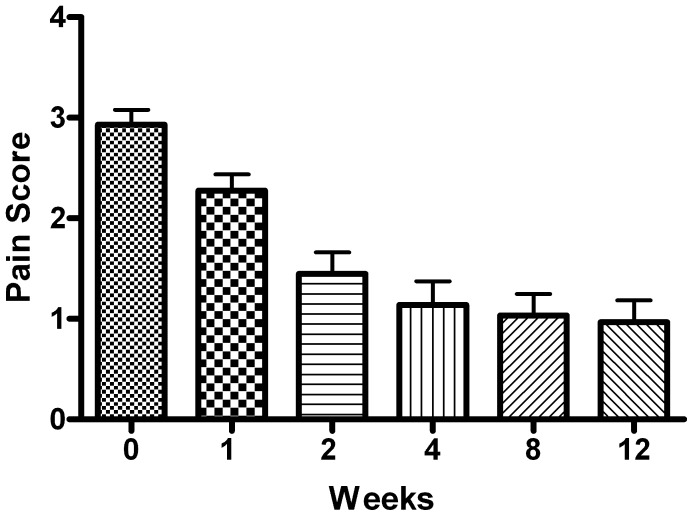
Pain Score as function of time. Pain score (mean ± SE), before treatment and at 1, 2, 4, 8 and 12 weeks from the beginning of therapy. Pain score ranges from 0 to 4, where 0 points denotes no pain, 4 points denotes severe pain.

Mean (±SED) 5 steps pain score before the onset of treatment was 2.93±0.8. It decreased to 2.28±0.88 after one week of treatment and then further to 1.45±1.15 after 2 weeks and to 1.14±1.27 after 4 weeks (Figure 2). The difference in mean score between each time point and the subsequent one was statistically significant (p<0.002 from treatment onset to week 1; p<0.0001 from week 1 to week 2, and p<0.01 from week 2 to week 4). At 8 weeks, the mean score measured 1.03±1.15, and at 12 weeks, 0.97±1.18 (Figure 2). The differences between weeks 4 and 8 (p = 0.45) and weeks 8 and 12 (p = 0.16) did not reach statistical significance. The decrease in pain from week 1 to week 12 was statistically significant (p<0.001).

**Figure 3:**
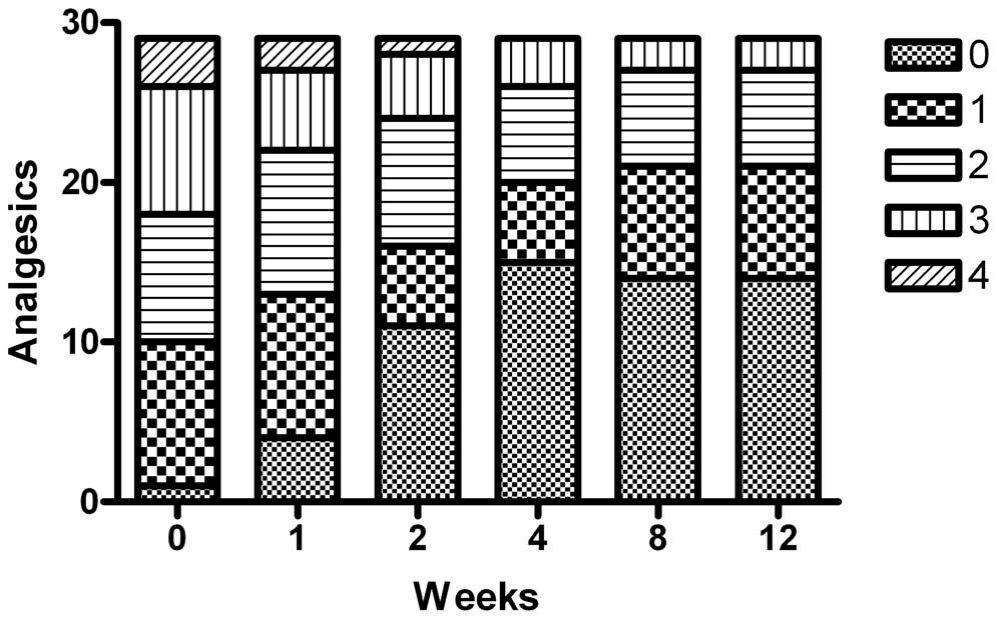
Analgesics score. Analgesics score at 1, 2, 4, 8 and 12 weeks from the beginning of treatment. Consumption of analgesics was evaluated by the physician on the basis of the medical records using the 5-point WHO score, as follows: level 0, no analgesics required; level 1, non-narcotic analgesics required occasionally; level 2, non-narcotic analgesics required regularly; level 3, narcotic analgesics required occasionally; level 4, narcotic analgesics required regularly. The decreases in analgesic score after 1, 2, and 4 weeks from the initiation of treatment were p<0.04, p<0.001 and p = 0.02, respectively.

Before initiation of treatment, an analgesics score of 0 was documented in 1 patient, a score of 1 in 9 patients, a score of 2 or 3 in 8 patients each, and a score of 4 in 3 patients. After 4 weeks, a score of 0 was documented in 15 patients, a score of 1 in 5 patients, a score of 2 in 6 patients, and a score of 3 in 3 patients, with no patient having a score of 4. Thus, the rate of low analgesics use (score 0 or 1) increased from 34% of patients before treatment to 55% after 2 weeks of treatment, to 69% (20/29) after 4 weeks, and to 72% (21/29) after 8 and 12 weeks. Like the pain score, the main changes in the analgesics score were noted during the first 4 weeks of treatment (p<0.04 from treatment onset to week 1; p<0.001 from week 1 to week 2; p = 0.02 from week 2 to week 4), with stabilization of the scores thereafter (p = 0.8 from week 4 to week 8, p = 0.4 from week 8 to week 12) (Figure 3).

### Response to Treatment

The clinical benefit of chemoradiation was determined on the basis of the pain and analgesics scores. The results are summarized in Table 3. PP was documented in two patients (7%) after one week and in one patient after 2, 4, and 8 weeks, and in none of the patients after 12 weeks. Fourteen patients (48%) had SP after one week, 6 (21%) at 2 weeks, 5 (17%) at 4 wks, 3 (10%) at 8 weeks, and 4 (14%) at 12 weeks. Four patients (14%) had a CR at 1 week, 11 (38%) at 2 weeks, 15 (52%) at 4 weeks, 15 (52%) at 8 weeks, and 14 (48%) at 12 weeks;. The corresponding figures for PR were 9 patients (31%) at 1 week, 11 (38%) at 2 weeks, 8 (28%) at 4 weeks, 10 (34%) at 8 weeks, and 11 (38%) at 12 weeks. The rate of overall response (OR), either CR or PR, increased over time and then stabilized: 45% at 1 week, 76% at 2 weeks, 79% at 3 weeks, and 86% at 8 and 12 weeks.

**Table 3:** Response to treatment with capecitabine and radiation.

Response	1 WK	2 WK	4 WK	8 WK	12 WK
CR	4 (14%)	11 (38%)	15 (52%)	15 (52%)	14 (48%)
PR	9 (31%)	11 (38%)	8 (28%)	10 (34%)	11 (38%)
SP	14 (48%)	6 (21%)	5 (17%)	3 (10%)	4 (14%)
PP	2 (7%)	1 (3%)	1 (3%)	1 (3%)	0 (0%)

Response to treatment was evaluated 1, 2, 4, 6, and 12 weeks (WK) after the initiation of chemoradiation. Complete response (CR), defined as no pain and no need for analgesics; partial response (PR), defined by a decrease of 2 points in the pain score and no change in analgesics consumption; stable pain (SP), defined as a decrease of one point or no change in the pain score and no change in analgesics consumption; progressive pain (PP), defined as an increase of 2 points in the pain score.

There was no difference in response between patients treated with bisphosphonates and those who did not receive these medications, and between patients with single versus multiple bone metastases. The reason for lack of differences probably stems from the high response rate to the combined chemo-radiotherapy in all groups on one hand and the small sample size on the other hand. These facts also limited us from preforming a multivariate analysis of the data.

## Discussion

This study examined the feasibility and safety of combining radiation therapy with capecitabine to palliate pain from bone metastases of breast cancer origin. Our results show that capecitabine and concurrent local radiation are well tolerated, with only mild side effects. The mean pain score decreased significantly from week 1 after treatment onset to week 12 (p<0.001), and also between successive time points early in the course of treatment. The pain relief was durable and was accompanied by a significant decrease in the need for pain- control medications. At 12 weeks from the onset of treatment, the OR rate was 86%; CR was achieved in 48% of patients and PR in 38%.

Response rates are difficult to compare among studies because the observed response is influenced by many factors, including the characteristics of the study population, the type of pain scale employed, the inclusion of quality of life as an endpoint, the consumption of analgesics, and the time to response determination. To help counter this problem, Chow et al. [29] published, in 2002, an International Consensus to standardize the criteria for response from radiotherapy to bone metastases. Three meta-analyses of randomized studies of various dose and time schedules of radiotherapy reported that the administration of single or multiple fractions for palliation of painful metastases yielded similar results [30], [31], [32]. Wu et al. [30] reported CR rates of 32% and 33% in the single- and multiple-fraction arms, respectively, and OR rates of 72% and 73%, respectively. However, rates were lower in the meta-analyses of Chow et al. [31] (OR, 58% and 59%; CR, 23% and 24%) and Sze et al. [32] (CR, 34% and 32%; OR, 60% and 59%).

We too applied the definitions of CR and PR recommended by the International Consensus, and we evaluated response at 12 weeks, as in the study of Chow et al. [29]. In recent randomized trials consensus definition of response was applied [33]–[35] which allows comparison with our results. Comparison of the present findings with the parallel arm in the RTOG study, in which the same radiotherapy dose was used [33], showed that our patients, receiving the combined therapy, experienced higher rates of both CR (48% vs 18%) and OR (86% vs 66%). Similarly, van der Linden et al. (34) reported lower response rates: CR rates of 13% for single-fraction therapy and 14% for multiple-fraction therapy, and OR rates of 68% and 69%, respectively. In contrast, while the randomized trial of Foro Arnalot et al. [35] yielded lower CR rates (11% and 13%) than the present study, the OR rate was similar. Altogether, with the limitations of cross-trial comparisons, it seems that the response rates observed in the current study, using combined chemoradiaton, are higher than those observed in earlier studies, using radiotherapy alone, at least concerning the CR rates.

The lack of difference in response rates between single- and multiple-fraction palliative radiation for bone metastases in all these studies supports the assumption that focusing on changes in dose fractionation will not significantly alter pain control. The results of the present study suggest that combining chemotherapy with radiotherapy may be a promising strategy to enhance the palliative effect of the later. Our review of the literature revealed no previous studies on this approach in this setting.

The present study was limited by a small sample size, non-comparative design, and the follow-up of patients was limited only to 12 weeks. Nevertheless, the results are encouraging, showing that combining capecitabine with irradiation for palliation of pain due to bone metastases in breast cancer patients is effective and well tolerated. Larger, randomized controlled phase III studies are needed to compare this approach to the standard treatment of radiotherapy alone, in this setting. Moreover, such studies may enable a comparison of the added value of the combined modality between patients with single bone metastasis and those with multiple skeletal lesions.

### Conclusion

This is the first report on the use of concurrent capecitabine and external beam radiotherapy for the treatment of bone metastases of any origin, and specifically of breast cancer origin. This study shows that this approach is safe and tolerable and results with an unusually high OR and CR rates, compared to those reported for radiotherapy alone. Further evaluation of this strategy is warranted.

## Supporting Information

Protocol S1 Trial Protocol.
(DOC)

Checklist S1 CONSORT Checklist.
(PDF)
