# Supplementary material for: Phase II Study of Concurrent Capecitabine and External Beam Radiotherapy for Pain Control of Bone Metastases of Breast Cancer Origin
Source: PLoS One. 2013 Jul 10;8(7):e68327. doi: 10.1371/journal.pone.0068327 (PMC3707893; doi:10.1371/journal.pone.0068327)
Supplement: Protocol S1 — Trial Protocol. (DOC) [file pone.0068327.s001.doc]

A PILOT STUDY OF CONCURRENT CAPECITABINE ( XELODA ) AND EXTERNAL BEAM RADIOTHERAPY FOR OSSEOUS METASTASES FROM BREAST CANCER.

BACKGROUND

Much of the clinical practice of oncology involves palliative care. In this setting ,the emphasis is on alleviation of symptoms and preservation or improvement of quality of life. A large body of clinical evidence documents the effectiveness of local-field external beam radiotherapy in palliation of pain from osseous metastases (1). Despite this general agreement, controversy remains regarding the optimal dose and fractionation schedule. Prospective phase III clinical trials (2-6), today, have failed to demonstrate superiority of one schedule over another ,and as a result, the patterns of practice of remain diverse in duration and intensity.

Between 1974 and 1980 the RTOG conducted a large national study to determine the effectiveness of five different dose fractionation schedules(2). A total of 1016 patients were entered ,266 into a “solitary metastasis” stratum, and 750 into a ”multiple metastasis “ stratum. The former were randomly assigned to treatment with 40,5Gy in 15 fractions or 20Gy in 5 fractions. The latter were assigned to 30Gy in 10 fractions, 15 Gy in5 fractions ,20 Gy in 5 fractions, or 25 Gy in 5 fractions. A quantitative measure of pain ,based on severity and frequency of pain, and the type and frequency of pain medications used, was devised to evaluate response. Overall, 89% of patients experienced minimal relief. There were no significant difference between the treatment arms in both strata. The initial pain score was found to be a useful predictor ; patients with high score were less likely to respond and were less likely to experience a complete response. Patients with breast and prostate cancer were significantly more likely to respond than patients with lung or other primary lesions. Patients completing their treatment as planned had significantly higher rates of complete response than those who did not .While some relief was experienced almost invariably within the first four weeks, complete relief was first reported later than four weeks after start of treatment in about 50 % of patients .The median duration of minimal and complete pain relief was 20 and 12 weeks ,respectively. There were no significant differences in duration of pain relief between the different arms It was concluded that all treatment dose schedules were equally effective.

A reanalysis of the RTOG study was reported by Blitzer (7).Using a stepwise logistic regression, he examined the effect of the number of fractions, the dose per fraction, and solitary versus multiple metastases, on the probability of attaining complete pain relief and the need for retreatment. This multivariate technique allowed patients with solitary and multiple metastases to be analyzed together. By increasing the number of subjects and events the statistical power of the analysis was outcome. There was no correlation of the time dose factor with outcome (8). It was concluded that the more protracted schedules resulted in improved pain relief.

Price et al. randomized 288 patients to receive either 8 Gy in one fraction or 30 Gy in 10 daily fractions. Pain was assessed using a questionnaire completed by the patients at the home on a daily basis. No differences were found in the probability of attaining pain relief, the speed of onset or the duration of relief between the two arms(4). Hoskin et al. randomized 270 patients to receive either 4 Gy or 8 Gy in one fraction (3) Pain assessed by the patient) and analgesic usage were recorded before treatment and at 2,4,8 and 12 weeks. At 4 weeks the response rates were 69% for 8 Gy and 44% for 4 Gy (p<0.001). The duration of the effect was independent of dose.

Two other randomized trials have been reported (5,6). Given the small difference in the Biological Effective Dose ( BED) between the arms (6) and the small number of patients accrued (5,6), it is not surprising that no differences between the treatment arms were detected.

RTOG have reported the results of a pooled data dose‑response analysis (11). A computerized literature search was conducted to identify prospectively randomized clinical studies which addressed this issue and the results of these studies (2‑6) were pooled together to form a database for analysis. The endpoint selected for analysis was complete response (CR). It was felt that this endpoint was most likely to be evaluated in a consistent fashion by different investigators. One study (6) was excluded from the analysis because outcome was not reported using conventional definitions of complete and partial response. To allow comparison of the different study arms, the BED was calculated for each schedule. Odds ratios calculated for various dose levels showed a statistically significant increase from 1.00 to 3.32 as the BED increased from 14.4 Gy to 5 1.4 Gy.

For the first time, RTOG analysis demonstrated a highly significant dose‑response relationship for palliation of pain from bone metastases with radiotherapy. Furthermore, there was no evidence of flattening of the dose­-response curve within the dose range tested, suggesting that further gains can be realized at doses outside of the range tested. We propose a phase II clinical trial in which the biological effective dose will be increased by the concurrent use of a radiosensitizer. The main advantage of this approach over escalation of the physical dose is the avoidance of increase in the overall treatment time.

Capecitabine is a fluoropyrimidine carbamate with antineoplastic activity. It is an orally administered systemic prodrug of 5'‑deoxy‑5‑fluorouridine (5'‑DFUR) which is converted to 5‑ Capecitabine is readily absorbed from the gastrointestinal tract. In the liver, a 60 kDa carboxyesterase hydrolyzes much of the compound to 5'‑deoxy‑5‑fluorocytidine (5'‑DFCR). Cytidine deaminase, an enzyme found in most tissues, including tumors, subsequently converts 5'‑DFCR to 5'‑deoxy‑5‑fluorouridine (5'‑DFUR).. The enzyme, thymidine phosphorylase (dThdPase), then hydrolyzes 5'‑DFUR to the active drug 5‑FU. Many tissues throughout the body express thymidine phosphorylase. Some human carcinomas express this enzyme in higher concentrations than surrounding normal tissues. Both normal and tumor cells metabolize 5‑FU to 5­fluoro‑2‑deoxyuridine monophosphate (FdUMP) and 5‑fluorouridine triphosphate (FUTP). These metabolites cause cell injury by two different mechanisms. First, HUMP and the folate cofactor, N5‑10­methylenetetrahydrofolate, bind to thymidylate synthase (TS) to form a covalently bound ternary complex. This binding inhibits the formation of thymidylate from uracil. Thymidylate is the necessary precursor of thymidine triphosphate, which is essential for the synthesis of DNA, so that a deficiency of this compound can inhibit cell division. Second, nuclear transcriptional enzymes can mistakenly incorporate FUTP in place of uridine triphosphate (UTP) during the synthesis of RNA. This metabolic error can interfere with RNA processing and protein synthesis. Capecitabine is already approved for use in patients with metastatic breast cancer resistant to both paclitaxel and an anthracycline‑containing chemotherapy regimen.

Although 5FU is an established radiosensitizer, the exact mechanism by which it enhances cell kill is not well understood. Sensitization is schedule dependent and is maximal when a cytocidal concentration of 5FU is given after the radiation exposure (12). The effect does not result from increased sublethal damage or inhibition of sublethal damage repair (12,13). Current evidence suggests that radiosensitization by 5FU is mediated through its effects on DNA rather then RNA (14). In vivo (15) as well as clinical studies (16) have demonstrated the superiority of continuous over bolus infusion of 5FU.

Over the last several decades, concurrent 5FU and radiation have been used successfully in a variety of malignancies. Several important lessons have been learned from clinical trials (16). Simultaneous delivery of large (IV bolus) doses of 5‑fluorouracil with irradiation is associated with improved survival compared to treatment with radiation alone, but at a price of increased normal tissue toxicity. These side effects can be ameliorated by using protracted venous infusion. This is an efficacious approach, with a wide therapeutic index, which permits concurrent treatment of micrometastatic disease and radiation sensitization.

Combined‑modality therapy, including irradiation and concurrently administered 5FU based chemotherapy, has become the mainstay of therapy for anal and rectal cancers. Protracted venous infusion chemoradiation is also used in the preoperative management of rectal cancer and in the nonoperative management of anal cancers. The Gastrointestinal Tumor Study Group (GITSG) has demonstrated a significant survival advantage for patients who receive adjuvant combined radiation and bolus 5FU following curative resection of pancreatic cancer (17,18). Similar advantages have been demonstrated in unresectable pancreatic cancers. Significant advantages have also been shown for concurrent chemotherapy regiments containing 5FU in esophageal cancer (19), squamous cell carcinoma of the Head and Neck (20,21), and carcinoma of the cervix (22).

There is very limited experience with the use of capecitabine and radiation. Based on its relative selectivity and the increase in tumor cell 5FU levels, it should be expected that capecitabine will provide superior radiosensitization at equivalent or reduced systemic toxicity levels**.** Interestingly, it has been recently reported that radiation induces thymidine phosphorylase and enhances the efficacy of capecitabine in human cancer xenografts (23). This may further enhance the synergistic effects, and consequently the therapeutic ratio.

In RTOG study a substantial number of patients with gastrointestinal malignancies treated with concurrent radiotherapy and capecitabine at a dose of 1600 mg/m2/day (5 days a week) with very little toxicity(24 ).In phase II study of chemoradiation for rectal cancer 1650mg/m2/d of capecitabine for 14 days was safe and well-tolerated treatment(25).

In this study we will investigate the feasibility of concurrent Capecitabine and external beam radiotherapy, as well as collect preliminary data regarding the efficacy of this regimen.

**OBJECTIVES**

Hypothesis: Given the hypothesis that regimens employing greater intensity radiation yield higher rates of pain relief, radiosensitization using a tumor targeted drug like Xeloda should improve the rate of complete pain relief as compared to radiosensitization with 5FU alone.

Primary Objective:

To determine the frequency and duration of pain relief and narcotic relief for the proposed regimen.

Secondary Objective:

To determine the toxicity of concurrent Capecitabine and radiotherapy in breast cancer patients with bone metastases.

**Eligibility Criteria**

Patients with metastatic breast will be allowed on the study regardless of their prior exposure to chemotherapy. Today, there is no evidence to suggest crossresistance between radiotherapy and prior chemotherapy, or that response to radiotherapy is related in any way to the number of lines of chemotherapy used in a particular patient. Good palliative responses can often be achieved in heavily pretreated patients.

1.The patient must be 18 years of age or older.

2.The patient must have histologically proven breast adenocarcinoma.

3. Radiographic evidence of bone metastasis is required .Acceptable studies include plain radiographs, radionuclide bone scans, computed tomography scans and magnetic resonance imaging.

4 .The patient must have pain that appears to be related to the radiographically documented metastasis.

5. Patients receiving systemic therapy with Capecitabine to metastatic disease (according to health basket ).

6. Patients must have an estimated life expectancy of 3 months or greater.

7. Patients will be eligible for treatment of multiple metastases only if these can be included in no more than two treatment sites.

8. Signed study‑specific informed consent.

9 Karnofsky Performance Status > 40.

10**.** Calculated Creatinine Clearance > 50 ml/min

11.ALT and AST no greater than 3‑5 times the institutional normal; bilirubin and serum creatinine no greater than 1.5 times normal; ANC greater than 1500, and platelets at least 100,00.

Exclusion Criteria

1. Prior radiation therapy or prior palliative surgery to the painful site.

2. Impending fracture of the treatment site or planned surgical fixation of the bone.

3. Patients with clinical or radiographic evidence of spinal cord or cauda equina compression.

4. Patients receiving systemic radionuclides (strontium, samarium, etc.) within 60 days prior to registration.

**PRETREATMENT EVALUATION**

1. Histologic diagnosis of the primary site.

2. Radiographic assessment, with must include plain x-ray of the index lesion (s) and a bone scane.

3. Pain assessment score (BPI ).

4. Laboratory studis within 2 weeks of registration (CBC, serum ALT, AST ,total bilirubin and creatinine).

# RADIATION THERAPY

1.Treatment must be given using 6-15MV photons or 6-18 MeV electrons .

2.All fields must be treated each day. Treatment volume will include the radiographic abnormality with at least a 2 cm margin. Treatment of the entire bone is not required.

3. Anterior and posterior parallel opposed fields will be used for lumbar spine, sacrum, and extremity sites. Equal weighting is recommended, although unequal weighting may be used for the lumbar or sacral spine with a ratio of doses of 1:2 AP:PA. Dose will be prescribed at mid‑depth at the central axis, or at the center of target volume if unequal weighting is used.

Alternatively, the lumbar spine may be treated with a single PA field, with the dose prescribed to the mid‑vertebral body as defined by a lateral simulator film.

4. Single posterior fields will be used for the thoracic spine and scapula .The treatment depth is set at the middle of the vertebral body, as determined by a lateral simulation film.

5. The cervical spine may be treated with either parallel opposed lateral fields or with a single posterior field. When lateral fields are used, the isocenter should be at mid‑thickness, with the dose prescribed to the mid‑vertebral body.

6. Pubic. bone lesions will be treated with a single anterior field at a depth determined by lateral radiograph or CT scan.

7. Clavicular lesions will be treated with a single anterior field at a depth of 3 cm. The dose will be prescribed to the 3 cm depth. An alternative depth may be used as determined by CT scan or other radiographs.

8. Rib metastases may be treated with electrons or with photons. When electrons are used, the appropriate energy should be chosen such that the entire lesion is covered by the 90% *(or higher)* isodose curve. The dose will be prescribed to the 100% isodose line. When photons are used, parallel opposed fields may be used, with the depth prescribed to the mid thickness. Tangential fields are strongly encouraged to avoid treatment of underlying structures. A single field may be used to cover the lesion, with the depth set at the estimated depth of the rib lesion, and the dose prescribed to that level.

9. When more than one osseous site is to be included into a treatment field, the treating radiation oncologist may use different field arrangements at herlhis discretion, with the goal of providing relatively uniform coverage of the target sites and minimum inclusion of uninvolved tissues.

**Radiation Dose**

All patients will receive radiotherapy to a dose 30 Gy in 10fractions (3 Gy per fraction) over two weeks.

**CHEMOTHERAPY**

Chemotherapy with capecitabine tablets will be given concurrently with radiotherapy in the dose 1400 mg/m2 orally, in two daily divided doses.

Total daily dose rounded to the nearest 500 mg and divided into morning and evening

Doses, as per the following table:

**Number of 500 mg tablets to be taken**

**Surface Area** Total Daily Dose **morning evening**

**(m2) (mg)***

<= 1.08 1000 1 1

1.09‑1.4 1500 2 1

1.41‑1.71 2000 2 2

1.72‑2.02 2500 3 2

***Total Daily Dose rounded to the nearest 500 mg and divided into**

**morning and evening doses.**

**Dose‑Limiting Toxicity**

i) >grade 3 non‑hematologic toxicity, except for diarrhea, nausea, vomiting, fatigue, anorexia, alopecia, fever and/or local reactions.

ii) grade 4 diarrhea lasting >3 days which is not controllable with loperamide or other such medications. Or grade 4 diarrhea that requires IV hydration.

iii) grade 4 neutropenia lasting >3 days

iv) grade 4 thrombocytopenia

v) grade 2 hand foot syndrome. Dose adjustments will be as per the following table:

| **Toxicity NCI Grades** | **During a Course of Therapy** |
| --- | --- |
| *Grade 1* | Maintain dose |
| *Grade 2* | |
| ‑ 1st appearance (non‑HFS) | Interrupt until resolved to grade 0‑1 |
| ‑ 1st appearance of HFS | Interrupt until resolved to grade 0‑1, then continue at 75 % of original dose. |
| ‑2nd appearance | Interrupt until resolved to grade 0‑1, then continue at 75 % of original dose. |
| ‑3rd appearance | Interrupt until resolved to grade 0‑1, then continue at 50 % of original dose. |
| ‑4th appearance | Discontinue treatment permanently |
| *Grade 3* | |
| ‑ 1st appearance | Interrupt until resolved to grade 0‑1, then continue at 75 % of original dose with prophylaxis where possible. |
| ‑2nd appearance | Interrupt until resolved to grade 0‑1, then continue at 50 % of original dose. |
| ‑3rd appearance | Discontinue treatment permanently |
| *Grade 4* | |
| ‑ 1st appearance | Discontinue permanently  or  If physician deems it to be in the patient's best interest to continue, interrupt until resolved to grade 0‑1, then continue at 50 % of original dose . |

Dosage modifications are not recommended for grade 1 events. Therapy with capecitabine should be interrupted upon the occurrence of a grade 2 or 3 adverse experience. Once the adverse event has resolved or decreased in intensity to grade 1, then therapy may be restarted at full dose or as adjusted according to the above table. If a grade 4 experience occurs, therapy should be discontinued or interrupted until resolved or decreased to grade 1, and therapy should be restarted at 50% of the original dose. Doses of capecitabine omitted for toxicity are not replaced or restored; instead the patient should resume the planned treatment cycles. Once the dose has been reduced it should not be increased at a later time.

## Toxicity

CAPECITABINE can induce diarrhea, sometimes severe. Patients with severe diarrhea should be carefully monitored and given fluid and electrolyte replacement if they become dehydrated. National Cancer Institute of Canada (NCIC grade 2 diarrhea is defined as an increase of 4 to 6 stools/day or nocturnal stools, grade 3 diarrhea as an increase of 7 to 9 stools/day or incontinence and malabsorption, and grade 4 diarrhea as an increase of >10 stools/day or grossly bloody diarrhea or the need for parenteral support. If grade 2, 3 or 4 diarrhea occurs, administration of capecitabine should be immediately interrupted until the diarrhea resolves or decreases in intensity to grade 1. Following grade 3 or 4 diarrhea, subsequent doses of capecitabine should be decreased. Standard antidiarrheal treatments (eg, loperamide) are recommended. Necrotizing enterocolitis (typhlitis) has also been reported.

Hand‑and‑foot syndrome (palmar‑plantar erythrodysesthesia or chemotherapy induced acral erythema) is characterized by the following: numbness, dysesthesia/paresthesia, tingling, painless or painful swelling, erythema, desquamation, blistering and severe pain. Grade 2 hand‑and‑foot syndrome is defined as painful erythema and swelling of the hands and/or feet and/or discomfort affecting the patient's activities of daily living. Grade 3 hand‑and­foot syndrome is defined as moist desquamation, ulceration, blistering and severe pain of the hands and/or feet and/or severe discomfort that causes the patient to be unable to work or perform activities of daily living. If grade 2 or 3 hand‑and‑foot syndrome occurs, administration of capecitabine should be interrupted until the event resolves or decreases in intensity to grade 1. Following the occurrence of grade 2 handand‑foot syndrome, subsequent doses of capecitabine should be decreased.

There has been cardiotoxicity associated with fluorinated pyrimidine therapy, including myocardial infarction, angina, dysrhythmias, cardiogenic shock, sudden death and electrocardiograph changes. These adverse events may be more common in patients with a prior history of coronary artery disease.

Patients with mild to moderate hepatic dysfunction due to liver metastases should be carefully monitored when capecitabine is administered. The effect of severe hepatic dysfunction on the disposition of capecitabine is not known. If drug related grade 2‑4 elevations in bilirubin occur, administration of capecitabine should be immediately interrupted until the hyperbilirubinernia resolves or decreases in intensity to grade 1. NCIC grade 2 hyperbilirubinernia is defined as 1.5 x normal, grade 3 hyperbilirubinemia as 1.5‑3 x normal and grade 4 hyperbilirubinemia as >3 x normal.

Increases in adverse events have been noted in patients with reduced renal function. Therefore, patients will be excluded for a calculated creatinine clearance of less than 50 ml/min.

Myelosuppression was rare.

**Assessment of Response**

Pain scores and narcotic scores will be determined using the guidelines in following table:

PAIN AND NARCOTIC CATEGORIES AND SCORES

PAIN ANALGESIA

Severity 0 ‑ No pain 0 ‑ None

I ‑ Mild I ‑ Analgesics (ASA, *Bufferin, Tylenol, Anacin, etc.)*

2 ‑ Moderate 2 ‑ Mild Narcotic, (< 112 gr. *codeine, Darvon, etc.)*

3 ‑ Severe 3 ‑ Moderate Narcotic (> 112 < gr. *codeine, Percoan, etc)*

4 ‑ Strong 4 ‑Narcotic, (> 1 gr. *codeine, demerol, morphine, etc)*

Frequency 0 ‑ None 0 ‑ None

I ‑ Occasional (< *daily)* I ‑ p.r.n. (< *daily)*

2 ‑ Intermittent *(at least daily)* 2 ‑ q.d. (I *tab. or cap./day)*

3 ‑ Frequent (>. I < 3 *daily)* 3 ‑ b.i.d. t.i.d. (> I < 4 *tab. or cap./day)*

4 ‑ Constant *(most of the time)* 4 ‑ > t.i.d. (> 4 *tab. or cap./day)*

Pain Score = Pain Severity Grade x Pain Frequency Grade

**Narcotic Score** = Analgesia Severity Grade x Analgesic Frequency Grade

Response will be evaluated by questionnaires at follow‑up visit at 2, 4 and 8 weeks after completion of radiotherapy and by phone call interviews (when necessary for completeness) in poor compliance patients.

The "worst pain score" will be used as response endpoint. The time to maximal pain relief is the time from the first day of irradiation to the time of the lowest pain score for average pain.

Response Definitions

**Complete response** is defined as an average pain score of 0 for two consecutive analysis periods. Narcotic consumption must not be increased. **Partial response**. A decrease of at least 2 points in the worst pain score for two consecutive analysis periods. Narcotic consumption must not be increased. **No response** (any of the following): A pain score that does not change within 8 weeks from the start of radiation therapy.

A 2 point increase in worst pain score that is sustained at a higher level in the month following the first day of radiation therapy.

A pain score that drops by at least 2 points and subsequent sustained rise *(on 2 successive questionnaires)* of pain score by at least 2 points. Any patient with progressive pain in the treated area should have plain radiographs of the area to assess for bone stability and pathologic fracture.

References.

1.

Hoskin PJ. Scientific and clinical aspects of radiotherapy in the relief of bone pain. Cancer Surv. 7:69, 1988.

2. Tong D, Gillick L, Hendrickson FR. The palliation of symptomatic osseous metastases: the

results of the Radiation Therapy Oncology Group. *Cancer* 1982; 50: 893.

3. Hoskin PJ, Price P, Easton D, et a]. A prospective randomized trial of 4 Gy or 8 Gy single doses

in the treatment of metastatic bone pain. Radiotherapy and Oncology 23:74‑78, 1992.

4. Price P, Hoskin PJ, Easton D, et al. Prospective randomized trial of single and multifraction

radiotherapy schedules in the treatment of painful bony metastases. Radiotherapy and Oncology

6: 247‑255, 1986.

Okawa T, Kita M, Goto M, et al. Randomized prospective clinical study of small, large and twice‑a‑day fraction radiotherapy for painful bone metastases. Radiotherapy and Oncology 13: 99‑104, 1988.

6. Madsen EL, Painful bone metastases: Efficacy of radiotherapy assessed by the patients: A

randomized trial comparing 4 Gy x 6 versus 10 Gy x 2. Int. J. Radiat. Oncol. Biol. Phys. 9:1775­

1779, 1983.

7. Blitzer PH. Reanalysis of the RTOG study of the palliation of symptomatic osseous metastasis.

*Cancer* 1985; 55: 1468‑1472.

8. Orton CG, Ellis F, A simplification in the use of the NSD concept in practical radiotherapy. Br.

J. Radiol. 1973; 46:529‑537.

9. Daut R, Cleeland C, Flanery R: Development of the Wisconsin Brief Pain questionnaire to assess

pain in cancer and other diseases. Pain 17:197‑210, 1983.

10.

11.

12.

13.

14.

Cella DF, Tulsky DS, Gray G, et al.: The Functional Assessment of Cancer Therapy Scale: Development and validation of the general measure. J Clin Oncol 11:570‑579, 1993.

Ben‑Josef E, Shamsa F, Youssef E, Porter AT. External beam radiotherapy for painful osseous metastases: pooled data dose response analysis. Int J Radiat Oncol Biol Phys 45(3):715‑719, 1999.

Byfield JE, calabro‑Jones P, Klisak 1, et al. Pharmacologic requirements for obtaining sensitization of human tumor cells in vitro to combined r‑fluorouracil or ftorafur and x‑rays. Int J Radiat Oncol Biol Phys 1982, 8:1923‑33.

Nakajima V, Miyamoto Y, Tanabe M, et al. Enhancement of mammalian cell killing by 5fluorouracil in combination with x‑rays. Cancer Res 39:3763‑3767, 1979.

Lawrence TS, Davis MA, Maybaum I Dependence of 5‑fluorouracil‑mediated radiosensitization on DNA‑directed effects. Int J Radiat Oncol Biol Phys 1994, 29(3):519‑23.

15. Weinberg MJ, Rauth AM. 5‑fluorouracil infusion and fractionated doses of radiation: Studies

with a murine sqamous cell carcinoma. Int J Radiat Oncol Biol Phys 13: 1691‑1699, 1987.

Rich TA. Infusional chemoradiation for rectal and anal cancers. Oncology (Huntingt) 1999, 13(10

Suppl 5):131‑4.

17. Gastrointestinal Tumor Study Group. Further evidence of effective adjuvant combined radiation

and chemotherapy following curative resection of pancreatic cancer. Cancer 1987, 59(12):2006­

10.

18. Kalser MH, Ellenberg SS. Pancreatic cancer. Adjuvant combined radiation and chemotherapy

following curative resection. Arch Surg 1985, 120(8):899‑903.

19. al‑Sarraf M, Martz K, Herskovic A, Leichman L, Brindle JS, Vaitkevicius VK, Cooper J, Byhardt

R, Davis L, Emami B. Progress report of combined chemoradiotherapy versus radiotherapy alone

in patients with esophageal cancer: an intergroup study. J Clin Oncol 1997, 15(l):277‑84.

20. Wendt TG, Grabenbauer GG, Rodel CM, Thiel HJ, Aydin H, Rohloff R, Wustrow TP, Iro H,

Popella C, Schalhorn A. Simultaneous radiochemotherapy versus radiotherapy alone in advanced

head and neck cancer: a randomized multicenter study. J Clin Oncol 1998, 16(4):1318‑24.

21. Al‑Sarraf M, LeBlanc M, Giri PG, Fu KK, Cooper J, Vuong T, Forastiere AA, Adams G, Sakr

WA, Schuller DE, Ensley JF. Chemoradiotherapy versus radiotherapy in patients with advanced

nasopharyngeal cancer: phase III randomized Intergroup study 0099. J Clin Oncol 1998,

16(4):1310‑7.

22. **Morris M, Eifel PJ,** Lu J, Grigsby PW, Levenback C, Stevens RE, Rotman M, Gershenson DM,

Mutch DG. Pelvic radiation with concurrent chemotherapy compared with pelvic and para‑aortic

radiation for high‑risk cervical cancer. N Engl J Med 1999, 340(15):1137‑43.

23. Sawada N, Ishikawa T, Sekiguchi F, Tanaka Y, Ishitsuka H. X‑ray irradiation induces thymidine

phosphorylase and enhances the efficacy of capecitabine in human cancer xenografts. Clin

Cancer Res 1999, 5(10):2948‑53.

24. U.N.Vaishampayan, E. Ben-Josef, P.A. Philip, V.K. Vaitkevicius, K.J. Levin and A.F. Shields. A single- institution experience with concurrent capecitabine and radiation therapy in gastrointestinal malignancies . Int J Radiat Oncol Biol Phys ,2002, 53,675-679.

25. Jun-Sang Kim, Jae-Sung Kim, Moon-June Cho and Wan-Hee Yoon .Preoperative chemoradiation using oral capecitabine in locally advanced rectal cancer. Int J Radiat Oncol Biol Phys ,2002,54,403-408. APPENDIX 1

SAMPLE **PATIENT CONSENT FORM**

**A PHASE II STUDY OF CONCURRENT XELODA (CAPECITABINE) AND EXTERNAL**

**BEAM RADIOTHERAPY FOR OSSEOUS METASTASES FROM BREAST CANCER**

Principal Investigator: Edgar Ben‑Josef, M.D.

In trod uction/Pu rpose:

1 am being asked to participate in a research study to evaluate the effectiveness of using radiation therapy combined with chemotherapy for bone pain. This study will help determine whether daily radiation therapy given in combination with a daily radiosensitizer drug Xeloda (capecitabine) better controls or eliminates the pain. 1 have the opportunity to decide whether to undergo the procedure after knowing the risks, benefits, and alternatives.

It has been explained to me that 1 have a tumor that has spread to my bones, which is causing pain. Radiation therapy alone is a frequently used treatment for this type of problem. Xeloda is an approved drug used to treat metastatic breast cancer when it becomes resistant to other chemotherapy. Studies have shown that Xeloda has radiosensitzer effects, making the tumor cells more sensitive to radiation therapy. This study will also determine any possible toxic effects of the combination treatment and see how the treatment affects the quality of my life.

**Procedure:**

This treatment to be given is as follows:

1 will receive radiation treatment to the painful area once a day. I will have five treatments a week, Monday thru Friday for fourteen to seventeen treatments. The amount of radiation given each day is 2.8 Gray per treatment. The radiosensitizer drug, Xeloda will be given daily during radiation therapy treatments, and 1 will take this by mouth.

Before, during and following the treatment, 1 will be asked to complete questionnaires regarding my pain and pain relief The questionnaires also ask questions about my overall quality of life and takes approximately 15‑20 minutes to complete. 1 will be asked to complete them before I start treatment and each time I see my doctor. Some of the questions regarding quality of life are of a personal nature, and may be upsetting to some patients. My doctor and nurse will be available discuss these questions if 1 have a concern or problem.

**Risks/Side Effects:**

Cancer treatment often have side effects. The treatments used in this program may cause all, some, or none of the side effects listed. In addition, there is always the risk of very uncommon or previously unknown side effects occurring.

Radiation therapy may cause hair loss in the area treated, dryness or irritation of skin in the area being treated *(like a sunburn or tanning),* and temporary tiredness. When certain areas of the body are treated, nausea, vomiting and sore throat, may occur. My blood counts may also be lowered causing tiredness or easy bruising. If my abdominal area is treated, I may have diarrhea, or feel as if my bladder is too full *(urgency)* causing me to urinate often. I can check with my doctor or nurse for more information on the side effects of my treatment.

Xeloda may cause frequent bowel movements or diarrhea. Hand‑and‑foot syndrome which is described as numbness, tingling, swelling, redness, and pain or no pain of palms and soles of the feet may occur. It can also cause heart conditions such as angina (chest pain), irregular heart beat especially with a prior history of heart disease. It can possibly lower the blood counts and increase the level of bilirubin in the blood.

My physician will be checking me closely to see if any of these side effects are occurring. Routine blood tests will be done to monitor the effects of treatment. Side effects usually disappear after the treatment is stopped. In the meantime, my doctor may prescribe medication to keep these side effects under control. The use of medication to help control side effects could result in added costs. This institution is not financially responsible for treatments of side effects caused by the study treatment.

If I am a sexually active women of child‑bearing potential I must take precautions to avoid pregnancy. It is known that this treatment could affect an unborn child. I will need to have a negative pregnancy test before entering this study. I will notify my doctor immediately if I become pregnant.

Benefits:

It is not possible to predict whether any personal benefit will result from the treatment program. A possible benefit would be some pain relief. The information learned from this study may be used scientifically and may possibly be helpful to others. Should my disease become worse, should side effects become very severe, should new scientific developments occur that indicate the treatment is not in my best interest, or should my physician feel that this treatment is no longer in my best interest, the treatment would be stopped. Further treatment would be discussed.

**Alternative Therapy:**

Alternatives that could be considered in my case include radiation therapy alone without being on this study to make me feel better. An additional alternative is no further therapy, which would probably result in continued pain and growth of my tumor.

**Confidentiality:**

Records of my progress while on the study will be kept in a confidential form at Wayne State University, Gershenson Radiation Oncology Center. During their required reviews, representatives of the Food and Drug Administration (FDA), the National Cancer Institute (NCI), qualified representatives of applicable drug manufacturers, and other groups or organizations that have a role in the conduct of this study may have access to medical records which contain my identity. However, no information by which I can be identified will be released or published.

**Compensation:**

In the event of any injury resulting from the research study, no reimbursement, compensation, or free medical care is offered by Karmanos Cancer Institute, Wayne State University, Gershenson Radiation Oncology Center, Harper Hospital, Sinai/Grace Hospital, Detroit Receiving Hospital, or the University Health Center.

**Cost of Participation:**

It has been made clear to me that costs associated with my participation in this study may not be covered

by my insurance carrier and the payment of such costs would be my responsibility. These costs may

possibly include side effect management resulting in hospitalization, follow‑up visits, labs, and/or

prescriptions. It has been sugges , ted that I contact my insurance carrier to determine its policy on payment

of cost of my participation in this research study.

Contact Persons/Questions:

In the event that injury occurs as a result of this research, treatment will be available. I understand, however, 1 will not be provided with reimbursement for medical care other than what my insurance carrier may provide nor will 1 receive other compensation. If 1 have any questions concerning my participation in this study now or in the future, Dr. Ben‑Josef, the investigator in charge, or one of his associates can be contacted at (313) 745‑9951.

If 1 have any questions regarding my rights as a research subject, 1 may contact the Chairman of the Human Investigation Committee at (313) 577‑1628.

**Voluntary Participation/Withdrawal:**

Participation in this study is voluntary. I am free to withdraw from this study at any time without prejudice to my subsequent care. 1 am free to seek care from a physician of my choice at any time. If 1 refuse to participate it will involve no penalty or loss of benefits or services to which 1 would otherwise be entitled.

**Consent to Participate in Research Study:**

The content and meaning of this information has been explained and is understood. All of my questions have been answered. 1 hereby consent and voluntarily offer to follow the study requirements and take part in the study. I will receive a signed copy of this consent form.

Patient's Name

(please print)

(Date)

(Signature of Patient or Legally Authorized Representative)

(Date)

(Date) Note: If there is anything that you do not understand about these explanations, please ask the doctor(s) for further information.

Witness

**PHYSICIAN'S STATEMENT**

1 have offered an opportunity to explain and give further details about this study to the individual whose signature appears on the above cited statement.

Signed:

Principal Investigator/Designee

(Date)

**APPENDIX 11**

**KARNOFSKY PERFORMANCE SCALE**

100 Normal; no complaints; no evidence of disease

90 Able to carry on normal activity; minor signs or symptoms of disease

80 Normal activity with effort; some sign or symptoms of disease

70 Cares for self; unable to carry on normal activity or do active work

60 Requires occasional assistance, but is able to care for most personal needs

50 Requires considerable.assistance and frequent medical care

40 Disabled; requires special care and assistance

30 Severely disabled; hospitalization is indicated, although death not imminent

20 Very sick; hospitalization necessary; active support treatment is necessary

10 Moribund; fatal processes progressing rapidly

0 Dead
